# The case for case–control studies in the field of suicide prevention

**DOI:** 10.1017/S2045796019000581

**Published:** 2019-10-01

**Authors:** Jane Pirkis, Angela Nicholas, David Gunnell

**Affiliations:** 1 Centre for Mental Health, Melbourne School of Population and Global Health, University of Melbourne, Parkville, VIC, Australia; 2 Population Health Sciences, Bristol Medical School, University of Bristol, Bristol, UK

**Keywords:** Epidemiology, research design and methods, risk factors, suicide

## Abstract

Much of our knowledge about the risk factors for suicide comes from case–control studies that either use a psychological autopsy approach or are nested within large register-based cohort studies. We would argue that case–control studies are appropriate in the context of a rare outcome like suicide, but there are issues with using this design. Some of these issues are common in psychological autopsy studies and relate to the selection of controls (e.g. selection bias caused by the use of controls who have died by other causes, rather than live controls) and the reliance on interviewing informants (e.g. recall bias caused by the loved ones of cases having thought about the events leading up to the suicide in considerable detail). Register-based studies can overcome some of these problems because they draw upon contain information that is routinely collected for administrative purposes and gathered in the same way for cases and controls. However, they face issues that mean that psychological autopsy studies will still sometimes be the study design of choice for investigating risk factors for suicide. Some countries, particularly low and middle income countries, don't have sophisticated population-based registers. Even where they do exist, there will be variable of interest that are not captured by them (e.g. acute stressful life events that may immediately precede a suicide death), or not captured in a comprehensive way (e.g. suicide attempts and mental illness that do not result in hospital admissions). Future studies of risk factors should be designed to progress knowledge in the field and overcome the problems with the existing studies, particularly those using a case–control design. The priority should be pinning down the risk factors that are amenable to modification or mitigation through interventions that can successfully be rolled out at scale.

As in other areas of public health, epidemiology has made major contributions to our understanding of the aetiology of suicide. Findings from studies of risk factors have helped shape preventive efforts and allowed us to prioritise them. Universal, selective and indicated interventions are all predicated on understanding and ameliorating risk among different target populations (Silverman and Felner, 1995; Silverman and Maris, 1995). In this editorial, we discuss some of the potential pitfalls in conducting certain types of epidemiological studies of suicide risk. We focus exclusively on studies where suicide is the outcome but note that studies of suicide attempts and suicidal thoughts face similar problems to those we highlight here, as well as some different ones.

Much of our knowledge about risk factors for suicide comes from two types of studies, psychological autopsy studies and register-based studies. Psychological autopsy studies involve collecting comprehensive information about the person who has died by suicide through interviews with those who were close to them (and, if possible, examination of coronial and medical records and other relevant documents) (Isometsa, 2001). Early psychological autopsy studies took the form of descriptive case series, with no control groups (Robins *et al*., 1959), but these days they tend to involve case–control designs, usually with living controls (Cavanagh *et al*., 2003; Pouliot and De Leo, 2006; Milner *et al*., 2012).

Register-based studies have routinely collected mortality data at their core. These typically link this mortality data to census data, and often to other datasets that record information on clinical factors (e.g. psychiatric diagnosis, use of mental health and general health services, or prescription of psychotropic or other medications) and social factors (e.g. educational attainment, welfare use, criminal justice involvement) that may confer risk. Some register-based studies follow the entire cohort over time, whereas others nest case–control studies within larger cohort studies (Erlangsen *et al*., 2018). We have conducted some examples ourselves (Pirkis *et al*., 2002; Chen *et al*., 2013), but much larger ones have emerged recently in countries such as the UK (John *et al*., 2014; Windfuhr *et al*., 2016), Sweden (Pethrus *et al*., 2017) and Taiwan (Weng *et al*., 2018).

Our focus for the remainder of this editorial is on case–control studies – both those that use the psychological autopsy method and those that are nested within larger cohort studies that involve population-based registers. Epidemiologists place case–control studies lower in the hierarchy of evidence than cohort studies. We would argue that case–control studies are appropriate in the context of a rare outcome and suicide is, fortunately, a relatively uncommon event, albeit a tragic one that often has huge ripple effects for families, friends and whole communities. Case–control studies are the best alternative design when cohort studies are challenging or simply not possible. Even when cohort studies are possible, embedding well-designed case–control studies within them may be a more efficient approach; Mortensen *et al*. (2000) led the way in doing this in the 1990s. Having said this, there are issues in conducting case–control studies to identify risk factors for suicide and we discuss these below.

One major issue relates to the selection of controls. The key thing about controls is that they should be drawn from the same population that gave rise to the cases and they would have been cases themselves if they had shown the outcome of interest, in this case, suicide (Wacholder *et al*., 1992). Some case–control studies in our field use people who have died of something else – often accidents – as controls. This approach is most common in psychological autopsy studies and may sometimes be employed because investigators are trying to rule out some of the informant-based differences between cases and controls that we describe below. Selecting controls who have died by accidents is problematic because observed differences are likely to be smaller, or non-existent, because dead controls have a heightened risk of premature death themselves.

To illustrate, socio-economic status (SES) will be a risk factor for suicide but also for accidental death, because most causes of premature death are socially patterned, so any study investigating socio-economic factors associated with suicide risk is likely to underestimate or eliminate effects if dead controls are used. For example, Palacio *et al*. (2007) conducted a psychological autopsy study which found no difference between those who died by suicide and those who died by accidents in terms of their odds of being unemployed. Gray *et al*. (2014) reported similar findings in a register-based study with psychological autopsy study components, observing no difference between those who died by suicide and those who died by accidents or from undetermined causes in terms of their likelihood of experiencing financial difficulties. These findings contrast with studies that use live controls that show considerably increased risk of suicide among unemployed individuals (Mortensen *et al*., 2000; Kposowa, 2001).

A further issue with the use of controls who have died as a result of accidents is that some suicides may be misclassified as accidents because there are insufficient grounds for police or coroners to determine suicidal intent. This is a particular challenge with some methods of suicide (e.g. poisoning with medicines or illicit drugs). For example, in our recent study of the coroners' records of 240 deaths deemed to be by accident/misadventure, clinical review identified 131 (54.6%) as likely suicides (Gunnell *et al*., 2013).

Another set of issues relates particularly to case–control studies that use the psychological autopsy method because these rely on interviews with informants. For cases the interviews occur with those who have lost someone to suicide, whereas for controls the interviews may be with the individuals themselves. This introduces the potential for bias because bereaved individuals may have a heightened focus on factors that they believe may have been associated with their loved one's suicide, particularly the presence of factors indicative of mental illness (see below). Furthermore, their knowledge of his or her characteristics and experiences will be ‘one step removed’ from information obtained directly from the individual themselves. Case–control psychological autopsy studies have come under particular criticism for giving too much weight to mental illness as a risk factor for suicide for this reason (Hjelmeland and Knizek, 2017).

One way various investigators have tried to combat this problem is by conducting interviews with the family and friends of live controls, but there is an argument that this may have an impact on the veracity of information for controls as well as cases. Niu *et al*. (2018) put this to the test in a case–control psychological autopsy study that focused on loneliness as a risk factor for suicide among older people in rural China. They collected data on loneliness from controls themselves and from proxies (next-of-kin, friends, neighbours and relatives) for these controls and found that there was only ‘fair’ agreement between the two.

There are other issues with interviewing proxies for controls too. Recall bias is an issue because the loved ones of cases will have thought about the events leading up to the suicide in some detail, trying to make sense of them in the knowledge that the person has died by suicide. They see these events through the lens of people bereaved by suicide, which is not the case for informants who are interviewed about controls.

Another difficulty lies in recruitment of both controls and their potential proxies. People who have been bereaved by suicide are often quite motivated to take part in research, viewing it as cathartic and hoping that it may prevent others having to experience what they have been through (Andriessen *et al*., 2018). By contrast, there may be less incentive for live controls to participate, and arguably less still for proxies for controls. Appleby *et al*. (1999) struck this issue and noted it as a limitation in their early case–control psychological autopsy study of suicides among young people in the UK. Their controls were identified from the practice registers of their cases' general practitioners, and each control was invited to nominate a family member or friend who could act as an informant. Of 286 potential controls, only 64 (22.4%) agreed to participate in the study and nominate an informant. The non-response issue is particularly problematic because it almost certainly introduces selection bias. We know from elsewhere in epidemiological research that response is associated with SES (Lorant *et al*., 2007), so differential response rates are likely to result in controls (and their proxies) being more educated, more likely to be employed and more affluent than cases (and their proxies).

Register-based studies can overcome some of the above problems because the registers they draw upon contain information that is routinely collected for administrative purposes and gathered in the same way for cases and controls. However, they face issues that mean that psychological autopsy studies will still sometimes be the study design of choice for investigating risk factors for suicide. Good quality register-based studies require sophisticated population-based registers that can be linked at the individual level, and many countries – particularly low and middle income countries – do not have these.

Even where comprehensive, high quality registers do exist, there will be variables that are of interest as potential risk factors for suicide that are not captured by them. For example, acute stressful life events that may immediately precede a suicide death (e.g. relationship breakdown, bankruptcy, being bullied, exposure to suicide-related news) are not routinely recorded in registers. It is also beyond the capacity of registers to capture variables like membership of certain population groups (e.g. LGBTI people), personality based factors (e.g. impulsivity, aggression, poor problem-solving skills) and environmental factors (e.g. access to means), all of which may confer risk for suicide.

Other variables of interest may be captured by registers but not in a comprehensive way. A case in point is register-based studies that examine the association between suicide attempts or mental illness and suicide, using inpatient admissions as evidence of the former. Many people who have made a suicide attempt or have mental health problems do not seek or receive care and are not admitted to hospital. For example, in the UK only around half of all people who present to hospital following a suicide attempt are admitted to a hospital bed (Cooper *et al*., 2013).

We would argue that future studies of risk factors should be designed to progress knowledge in the field and overcome the problems with existing studies, particularly those that use a case–control design. There will be mileage in developing methods for capturing risk factors in what is often a short transition period between suicidal thoughts and suicide attempts using smartphone and ecological momentary assessment approaches (Coppersmith *et al*., 2019), but we acknowledge that this is difficult. It will be important to focus our attention on emerging risk factors that may account for changing trends in suicide in different countries (e.g. the rise in youth suicide in various high income countries), rather than on well-established risk factors. An increased emphasis on the interaction between risk factors and individuals' susceptibility would also be desirable, as would a greater emphasis on protective factors. There may also be benefits in developing clever approaches that enrich and validate psychological autopsy data with register-based data, taking the best features of each. In all of these activities, the priority should be pinning down the risk factors that are amenable to modification or mitigation through interventions that can be successfully rolled out at scale.
